# On microbial virulence, mammals, and climate change

**DOI:** 10.25646/10391

**Published:** 2022-08-31

**Authors:** Arturo Casadevall

**Affiliations:** Johns Hopkins School of Public Health, Baltimore, USA

Virulence is a microbial property that is expressed only in a susceptible host. This raises interesting evolutionary questions: Why are some microbes pathogenic while the majority is harmless? Are pathogenic and non-pathogenic microbes different? How does virulence emerge in environmental microbes that are pathogenic despite having no need for their hosts? Answering these questions requires new conceptual tools. The damage-response framework developed more than two decades ago posits that there are only microbes and hosts and that what really matters is the outcome of interaction. This framework argues that the states of commensalism, symbiosis, latency and disease are all continuous and differ only in the amount of damage incurred by the host from the interaction. To create a quantitative view of virulence, the pathogenic potential concept was developed, which states that all microbes have some inherent pathogenic potential such that host immunity can usually be overcome with large inocula.

The fungal kingdom, with its tremendous diversity, provides insight into potential answers. Of the more than 1.5 million fungal species only about 150 to 300 are pathogenic for humans, and of these, only 10 to 15 are relatively common pathogens. In contrast to the paucity of fungal pathogens of mammals, fungi are major pathogens for plants and insects. Analysis of thermal tolerance in fungi suggests that vertebrate endothermy and homeothermy create a restricted environment for most fungal species. Hence, the combination of vertebrate adaptive immunity with endothermy probably accounts for the remarkable resistance of mammals to fungi [1].

The resistance of mammals to fungal disease based on endothermy in turn raises the question of how such an energetically unfavourable lifestyle was selected for in evolutionary history. There is evidence in the geologic record for massive proliferation of fungi at the end of the Permian and Cretaceous geologic epochs. Fungi, as degraders of organic matter, thrive in conditions of global catastrophe and the fungal proliferation following the bolide that ended the Cretaceous period would have created massive numbers of spores that would have greatly increased the likelihood of fungal disease for any survivors of the catastrophe. The author presented the hypothesis that fungal diseases contributed to both the extinctions at the end of the Cretaceous that resulted in the demise of the dinosaurs and to the great mammalian radiation that followed in the Tertiary era. Hence, the argument goes that mammals are resistant to fungal disease because they were preferentially selected to survive in the post-impact world due to their endothermy [2, 3].

Finally, the seminar considered possible consequences of climate change, which include the emergence of new fungal diseases as fungal species adapt to a warmer world. The emergence of *Candida auris* could represent the first example of this new threat.
